# Current Diagnostic and Therapeutic Aspects of Eosinophilic Myocarditis

**DOI:** 10.1155/2016/2829583

**Published:** 2016-01-17

**Authors:** Petr Kuchynka, Tomas Palecek, Martin Masek, Vladimir Cerny, Lukas Lambert, Ivana Vitkova, Ales Linhart

**Affiliations:** ^1^2nd Department of Medicine-Department of Cardiovascular Medicine, First Faculty of Medicine, Charles University in Prague and General University Hospital in Prague, U Nemocnice 2, 128 08 Prague 2, Czech Republic; ^2^Department of Radiology, First Faculty of Medicine, Charles University in Prague and General University Hospital in Prague, U Nemocnice 2, 128 08 Prague 2, Czech Republic; ^3^Institute of Pathology, First Faculty of Medicine, Charles University in Prague and General University Hospital in Prague, U Nemocnice 2, 128 08 Prague 2, Czech Republic

## Abstract

Eosinophilic myocarditis (EM) represents a rare form of myocardial inflammation with very heterogeneous aetiology. In developed countries, the most prevalent causes of EM are hypersensitivity or allergic reactions, as well as hematological diseases leading to eosinophilia. The disease may have a variable clinical presentation, ranging from asymptomatic forms to life-threatening conditions. Most patients with EM have marked eosinophilia in peripheral blood. Endomyocardial biopsy needs to be performed in most cases in order to establish a definitive diagnosis of EM. The therapy depends on the underlying aetiology. Immunosuppressive therapy represents the treatment mainstay in the majority of EM forms.

## 1. Introduction

The association between blood eosinophilia and related cardiac pathology was first documented in 1936 by Löffler, who described two cases of endocarditis parietalis fibroplastica [1]. Since that time much research has focused on eosinophilic heart disease. Eosinophilic myocarditis (EM) represents the initial stage of cardiac disorder that can disappear with or without any sequelae or may lead to advanced heart disease characterized by endomyocardial fibrosis.

## 2. Eosinophils

Eosinophils, along with other polymorphonuclear leukocytes, are produced by the bone marrow. They gradually differentiate into mature eosinophils under the influence of several cytokines. This maturation process takes approximately eight days. The main cytokines responsible for increases in eosinophil numbers are granulocyte macrophage colony-stimulating factor, interleukin- (IL-) 3, and IL-5 [2]. Among these cytokines, IL-5 produced by T helper 2 T lymphocytes is considered to be the major eosinophil growth factor. Moreover, this cytokine is also involved in survival, chemotaxis, and degranulation of eosinophils. These cells usually remain in the peripheral blood for only 8–12 hours before migrating to certain tissues. Extravasation of eosinophils from the bloodstream is considered to be a dynamic multistep process that involves capture, rolling, activation, adhesion, and transendothelial and subendothelial migration of the cells. In this process preactivation of eosinophils mediated by P-selectin and IL-5 seems to very important. In healthy subjects, eosinophils are normally found in the blood and in certain tissues (e.g., all portions of gastrointestinal tract with the exception of the oesophagus) [3]. The upper normal limit of eosinophils in the peripheral blood is 3–5% with a corresponding absolute eosinophil count of 350–500/mm^3^. The severity of eosinophilia has been arbitrarily divided into mild (<1500/mm^3^), moderate (1500–5000/mm^3^), and severe (>5000/mm^3^) [4]. Eosinophils measure 12–15 *μ*m in diameter and are characterized by a bilobed nucleus and numerous eosin-staining granules in their cytoplasm. These granules contain high concentrations of hydrolases, cationic and basic proteins. The most important cationic proteins are major basic protein (MBP), eosinophil cationic protein (ECP), eosinophil-derived neurotoxin, and eosinophil peroxidase. These proteins can lead to production of free radicals and induction of cell apoptosis or necrosis. Eosinophils are involved in the process of inflammation, as well as innate and adaptive immunity. Their specific granules are capable of inducing tissue damage and dysfunction by degranulation following activation by an immune stimulus [3].

## 3. Prevalence of Eosinophilic Myocarditis

Eosinophilic myocarditis is a rare myocardial disease. This form of myocarditis has been identified in 0.5% of cases in an unselected autopsy series [5] and in 0.1% of cases among a cohort of patients biopsied for suspected myocarditis [6]. The prevalence of EM in patients undergoing heart transplantation differs among published studies; usually it is reported between 3 and 7% [7, 8].

## 4. Pathophysiology of Eosinophilic Myocarditis

Eosinophilic heart disease includes several types of cardiac damage from acute myocarditis to endomyocardial fibrosis. The degree of heart involvement associated with eosinophilic infiltration of cardiac tissue depends on the stimulus attracting the eosinophils, the duration of eosinophilia, and the degree of eosinophil activation. Deleterious effects are more common in subjects with profound blood eosinophilia (>5000/mm^3^) [1]. Three phases of eosinophilic heart disease are classically described. The first stage represented by EM is due to initial eosinophilic infiltration of the heart and subsequent myocardial necrosis associated with the degranulation of eosinophils. When endomyocardial biopsies are performed, deposits of ECP, MBP, and eosinophil peroxidase have been consistently detected [1]. The second phase, known as the thrombotic stage, is mainly associated with a hypercoagulable state associated with increased levels of circulating thrombin. Because eosinophil cationic proteins normally bind to an anionic exosite on thrombomodulin, higher numbers of circulating eosinophils bind available thrombomodulin, causing impaired formation of the thrombomodulin-thrombin complex. Moreover, eosinophils store tissue factor, the main initiator of blood coagulation, in their specific granules. Furthermore, it has been recently shown that tissue factor expression is higher in subjects with hypereosinophilia [9]. The third and final phase of EM is represented by fibrotic scarring. Eosinophil-associated fibrosis is observed specifically in the endocardium because endothelial cells are very sensitive to eosinophil granule constituents, especially to ECP and MBP. Eosinophils have the potential to promote fibroblast activation, proliferation, and extracellular matrix production, likely through secretion of transforming growth factor- (TGF-) *β* and IL-1 [2, 3].

## 5. Aetiology of Eosinophilic Myocarditis

The principal aetiologic factors associated with EM are hypersensitivity or allergic reactions, infections, malignancies, vasculitis, and hypereosinophilic syndromes. In developed countries, EM seems to be predominantly connected with hypersensitivity or allergic reactions due to various stimuli including drug reactions. Drugs that are most frequently associated with EM are listed as follows [10].

*Principal Drugs Associated with Eosinophilic Myocarditis (Based on Table 3 in [10])*. Consider the following:

 
*Antimicrobial drugs* (amphotericin B, ampicillin, chloramphenicol, penicillin, tetracycline, streptomycin, cephalosporin, sulfonamides, and antituberculous drugs).
 
*Antipsychotics* (clozapine).
 
*Anti-inflammatory drugs* (indomethacin, oxyphenbutazone, and phenylbutazone).
 
*Diuretics* (acetazolamide, chlorthalidone, hydrochlorothiazide, and spironolactone).
 
*Angiotensin converting enzyme inhibitors* (captopril, enalapril).
 
*Inotropes* (dobutamine, digoxin).
 
*Others* (tetanus toxoid, methyldopa, amitriptyline, lenalidomide, and sulfonylurea).

In patients undergoing heart transplantation, EM is occasionally observed as an incidental histological finding in endomyocardial biopsy (EMB) specimens before heart transplantation, as well as in explanted heart specimens obtained at the time of transplantation. There may be an association between EM and dobutamine use, particularly prolonged intravenous administration [11].

Eosinophilia may be associated with a number of neoplastic disorders. It is considered to be reactive in some solid lung, GIT, and urogenital tumors as well as in certain types of hematologic disorders such as T-cell and Hodgkin lymphomas, acute lymphoblastic leukemia, or mastocytosis. Eosinophilia can also be part of the neoplastic clone in hematologic disorders, such as in acute and chronic myeloid leukemia, myelodysplastic syndrome, or other myeloproliferative diseases including polycythemia vera or essential thrombocythemia [12]. Reactive eosinophilia can be associated with various microbial agents but it usually represents a sequela of parasitic infections. Protozoal infections caused by* Trypanosoma*,* Toxoplasma*,* Trichinella*,* Entamoeba*, or* Echinococcus* are usually among the reported infectious causes of EM [10].

Eosinophilic myocarditis may develop in individuals suffering from certain types of vasculitis, namely, Churg-Strauss syndrome (CSS). This rare entity is also known as eosinophilic granulomatosis with polyangiitis. The syndrome was first described by Churg and Strauss as a disease characterized by disseminated necrotizing vasculitis with extravascular granulomas occurring among patients with bronchial asthma and tissue eosinophilia. Currently, diagnosis of CSS is based on criteria described by the American College of Rheumatology [13]. According to this classification at least four of the following criteria must be met for diagnosis of CSS: (1) marked peripheral eosinophilia >10%, (2) paranasal sinus abnormality, (3) bronchial asthma, (4) nonfixed pulmonary infiltrates, (5) mononeuropathy or polyneuropathy, and (6) extravascular eosinophil infiltration on biopsy findings. Heart involvement is more common in the subgroup of CSS patients with absence of ANCA (anti-neutrophil cytoplasmic antibody) [13]. Symptomatic cardiac manifestations occur in 27–47% of CSS cases and represent the major cause of death and poor long-term prognosis in these individuals [14].

Eosinophilic myocarditis can be also caused by a heterogeneous group of hematologic disorders called idiopathic hypereosinophilic syndrome (HES). This rare condition is defined as unexplained peripheral blood eosinophilia (>1500/mm^3^) persisting for at least six months associated with tissue damage [3]. If tissue damage is absent, idiopathic hypereosinophilia is the preferred diagnosis. Dermatologic involvement followed by pulmonary and gastrointestinal manifestations is most common in HES. Heart involvement occurs in approximately 20% of patients with HES and only a minority has cardiac manifestations at the time of initial presentation [15].

## 6. Clinical Manifestation

Eosinophilic myocarditis may present in many different ways, ranging from asymptomatic cases to life-threatening conditions such as cardiogenic shock or sudden cardiac death due to malignant ventricular arrhythmias. The diversity of clinical scenarios depends also on the underlying cause of eosinophilia. Prior to the onset of EM, approximately two-thirds of patients have symptoms of the common cold and one-third of cases suffer from allergic diseases such as bronchial asthma, rhinitis, or urticaria [16]. The manifestation of EM, similarly as in other types of myocarditis, may be in the form of chest pain, dyspnoea, fatigue, palpitations, or syncope.

## 7. Laboratory Markers

Eosinophilia in peripheral blood samples is present in the vast majority of patients with EM and is very useful in the diagnosis of EM. Essentially, the finding of hypereosinophilia in a patient presenting with cardiac symptoms should always raise high suspicion for EM. However, peripheral eosinophilia may be absent in the early stage of EM and may not develop during the course of the illness in a small subgroup of affected individuals.

Inflammatory markers like C reactive protein levels and erythrocyte sedimentation rate as well as levels of markers of myocardial injury such as creatine kinase or troponins are often raised in EM, but their absence does not exclude myocarditis. Moreover, their elevation is not specific for myocarditis. This also applies to brain natriuretic peptides, circulating cytokines, markers related to extracellular matrix degradation, and new biomarkers such as pentraxin, galectin, and growth differentiation factor [17].

## 8. Electrocardiogram (ECG)

An electrocardiogram is one of the first-line tests for suspected myocarditis. Although the ECG is often abnormal in EM, mostly demonstrating ST-T segment abnormalities, ECG signs are neither sufficiently specific nor sensitive for myocarditis [17]. Nevertheless, some ECG features like QRS complex prolongation are known to be associated with poor clinical outcome [17].

## 9. Echocardiography

Echocardiography is a very useful first-line method in diagnosis of EM. It helps not only to rule out other causes of patients' complaints but also to assess and monitor changes in cardiac chamber size, wall thickness, and ventricular systolic and diastolic function and to detect the presence of pericardial effusion and observe its dynamics. As mentioned above, cardiac involvement associated with hypereosinophilia is classified into three stages based on the degree of eosinophil-mediated heart injury. The first stage, known as necrotic stage, corresponds typically to EM and there are no pathognomonic echocardiographic signs that reliably distinguish between EM and other types of myocarditis [18]. In fulminant cases of myocarditis, a nondilated, thickened, and hypocontractile left ventricle (LV) is usually observed. However, a wide range of echocardiographic features may be present in patients with nonfulminant EM, ranging from severe global LV systolic dysfunction to almost normal echocardiographic findings. During the next thrombotic stage, endomyocardial and valvular involvement occurs, with the possibility of thrombus formation in the apical parts of the ventricles. Finally, at the last fibrotic stage, endomyocardial scarring progresses and restrictive cardiomyopathy develops. In an echocardiographic study by Ommen et al. which included 51 patients with idiopathic HES and assessed the degree of cardiac involvement, endocardial thickening was present in 12% of the subjects, posterior mitral valve leaflet involvement in 20%, tricuspid involvement in 10%, LV hypertrophy in 10%, LV dilatation in 14%, LV apical thrombus in 24%, and right ventricle (RV) apical thrombus in 20% [19].

## 10. Cardiac Magnetic Resonance (CMR)

**Figure 1:**
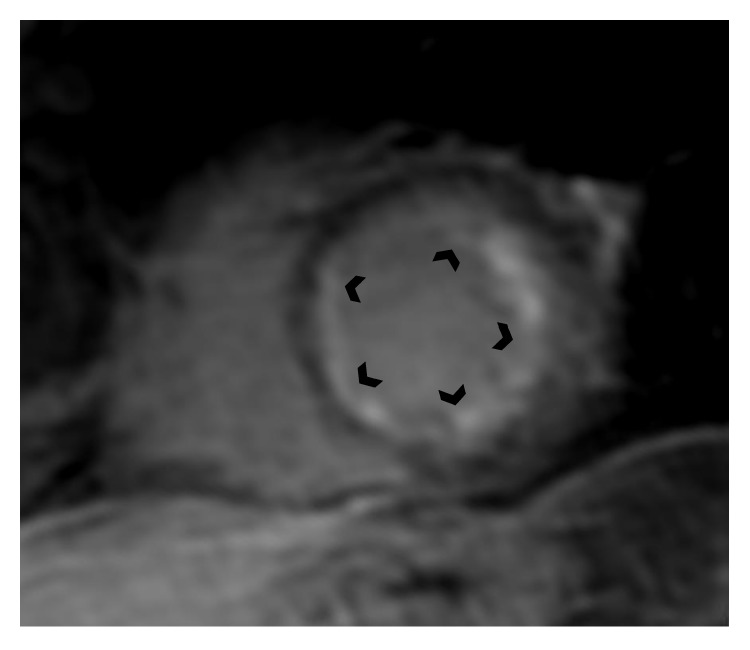
Cardiac magnetic resonance (short-axis) showing global subendocardial late gadolinium enhancement of the left ventricle in a patient with histologically proven eosinophilic myocarditis.

Cardiac magnetic resonance is currently the gold standard in noninvasive diagnosis of myocarditis. Its main advantage with respect to diagnosis of myocardial inflammation is its availability to characterize myocardial tissue. Based on the generally accepted Lake Louise criteria [20], a CMR study is consistent with the presence of myocarditis if at least two of the three following criteria are present: (1) regional or global myocardial signal increase in T2-weighted images, (2) increased global myocardial early gadolinium enhancement ratio between myocardium and skeletal muscle in gadolinium enhanced T1-weighted images, and (3) at least one focal lesion with nonischemic regional distribution in inversion recovery-prepared late gadolinium enhanced T1-weighted images. The presence of LV systolic dysfunction or pericardial effusion provides supportive evidence for myocarditis. In contrast to other types of myocarditis, EM is often associated with subendocardial late gadolinium enhancement, which can be patchy or diffuse (Figure 1). As opposed to ischemic heart disease, these subendocardial regions of late gadolinium enhancement in EM are not restricted to the territory of one of the main coronary arteries [21]. Moreover, in more advanced stages of heart involvement associated with eosinophilia, endomyocardial fibrosis with typical apical LV or RV involvement can be easily detected by CMR.

## 11. Cardiac CT and PET-CT

**Figure 2:**
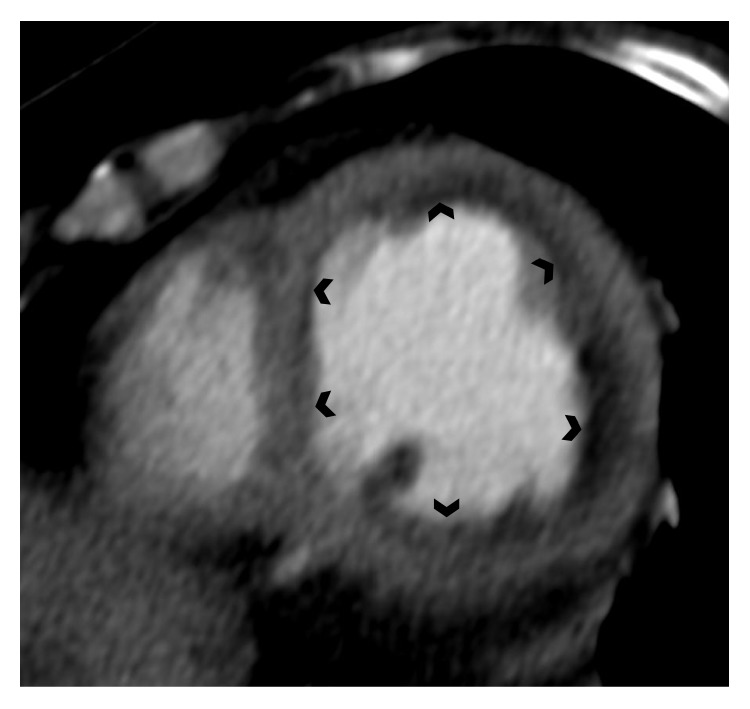
Contrast enhanced CT scan demonstrating diffuse subendocardial hypodensity of the left ventricle in a patient with histologically proven eosinophilic myocarditis.

Cardiac CT can be useful mainly to exclude significant coronary artery disease in patients with EM presenting with chest pain. Moreover, in those who are unable to undergo CMR it might represent an alternative method for noninvasive detection of myocarditis (Figure 2). PET-CT plays an important role in assessing the activity of the underlying disorder causing hypereosinophilia. In CSS, PET-CT examination is able not only to detect myocardial involvement but also to distinguish between myocardial fibrotic and inflammatory lesions [22].

## 12. Endomyocardial Biopsy

**Figure 3:**
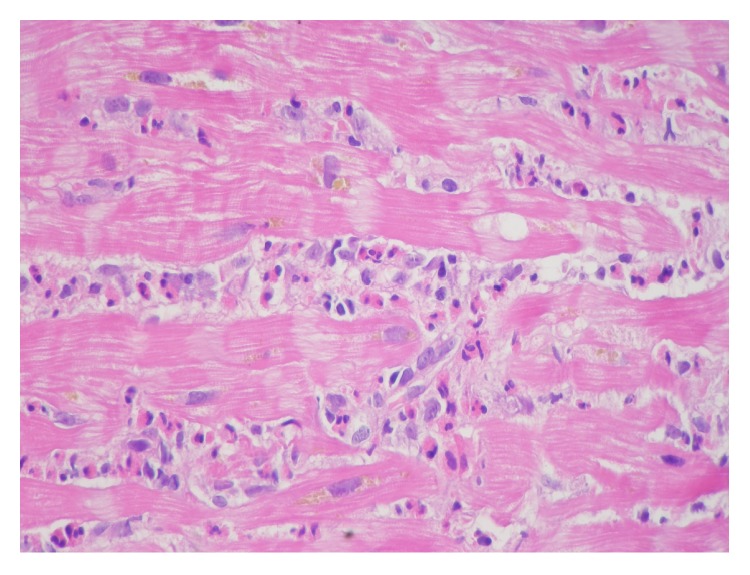
Endomyocardial biopsy demonstrating eosinophilic myocarditis (hematoxylin-eosin, magnification 600x).

Endomyocardial biopsy is currently the only method which can make the definite diagnosis of EM by confirming eosinophilic infiltration of the myocardium (Figure 3). In cases of focal myocarditis and less profound eosinophilic myocardial involvement, negative biopsy results may occur due to sampling error. However, if there is a strong clinical suspicion for EM, endomyocardial biopsy should be repeated.

## 13. Treatment

Generally, strict restriction of physical activity is recommended in all patients during the acute phase of EM with subsequent exclusion of sporting activities in next 6 months [17]. Pharmacological and nonpharmacological treatment of patients with EM manifesting with heart failure or arrhythmias are managed according to current guidelines.

Specific treatment of EM differs significantly based on its underlying aetiology. In patients with suspicion for hypersensitivity or allergic aetiology of EM, it is of utmost importance to eliminate possible causative factors. If EM is related to infectious agents such as parasites, targeted antimicrobial treatment is obviously essential. In myeloproliferative disorders associated with fusion genes FIP1L1 (FIP 1 like 1), PDGFRA (platelet derived growth factor receptor alpha), and PDGFRB (platelet derived growth factor receptor beta), respectively, tyrosine kinase activity is constitutively present. The administration of the tyrosine kinase inhibitor imatinib is clearly indicated in this situation. Oral treatment with imatinib can effectively suppress but not eliminate the FIP1L1-PDGFRA clone in most patients, although some may experience remissions after imatinib discontinuation [3]. In patients with EM, prophylactic use of steroids during the first days of imatinib treatment is recommended [3]. The majority of individuals with EM are treated with immunosuppressive treatment, namely, corticosteroids. Nevertheless, the evidence supporting this widely used therapy in non-CSS patients is modest and is based only on case reports, case series, and small nonrandomized studies [15]. Moreover, the initial dosage of corticosteroids and the treatment duration vary among the published studies and thus no clear evidence-based recommendations can be given at this time. It seems reasonable to adjust the dosage of corticosteroids and the treatment duration with respect to the severity of EM manifestation as well as the primary underlying disorder. In patients with CSS, corticosteroids are the mainstay of treatment. Patients with CSS are most frequently treated with 1 mg/kg per day of prednisone or its equivalent administered orally. When a clinical response is reached, usually in several weeks, steroids are tapered down slowly [13]. If a more advanced stage of the disease is present, combined immunosuppressive therapy comprising corticosteroids and cyclophosphamide or azathioprine is usually administered [13]. In a study conducted by Miszalski-Jamka et al., patients suffering from CCS in whom noncorticosteroid immunosuppressive treatment was initiated at the time of diagnosis less frequently had new onset or progression of heart failure in comparison with subjects in whom this therapy was started later on [23]. In patients with CSS or HES there is also evidence showing the usefulness of mepolizumab administration. Mepolizumab is a humanized monoclonal antibody that inhibits binding of IL-5 to its receptor expressed on eosinophils. Initial experience with mepolizumab demonstrates its safety and tolerability; a main advantage is its corticoid-sparing effect [24].

Interestingly, there is also evidence that certain patients with EM do not need to be treated with corticosteroids. In a retrospective study by Yanagisawa et al. [25], which included 22 patients with idiopathic eosinophilia and histologically proven EM and 7 subjects with lymphocytic myocarditis, a similar outcome in terms of LV ejection fraction improvement as well as mortality was observed at 1-year follow-up in both study groups with only conventional heart failure therapy.

Recently, new therapeutic strategies for eosinophil-associated disorders have been suggested. Among the plethora of eosinophils receptors described so far, only several receptors such as IL-5 receptor alpha, chemokine receptor CCR3, and sialic acid-binding immunoglobulin-like lectin 8 are considered to be relatively specific for eosinophil lineage and are thus potentially suitable for antibody targeting [26]. Among the drugs affecting these receptors benralizumab, representing humanized antibody to IL-5 receptor alpha, seems to be very promising and has evidence on blood and tissue eosinophilia reduction from several randomized studies [26]. Unfortunately, benralizumab similar to other new drugs targeting specifically eosinophil receptors lacks evidence from randomized trial for the treatment of EM.

## 14. Conclusions

Eosinophilic myocarditis is a rare myocardial disorder with heterogeneous aetiology. Peripheral blood eosinophilia associated with cardiac symptomatology should always raise suspicion for EM. Noninvasive imaging methods, namely, CMR, play an important role in the diagnostic process of EM. However, the definite diagnosis of EM usually needs to be confirmed by endomyocardial biopsy. Specific treatment of EM differs significantly based on its underlying aetiology. Immunosuppressive therapy represents the mainstay of treatment in the majority of patients with EM.
